# Frequency of gestational malaria and maternal–neonatal outcomes, in Northwestern Colombia 2009–2020

**DOI:** 10.1038/s41598-022-15011-1

**Published:** 2022-06-29

**Authors:** Jaiberth Antonio Cardona-Arias, Jaime Carmona-Fonseca

**Affiliations:** 1 Universidad de Antioquia UdeA, Calle 70 No. 52-21, Medellin, Colombia; 2 School of Medicine, University of Antioquia, Medellin, Colombia; 3 Coordinator of the Research Group “Salud y Comunidad César Uribe Piedrahita”, University of Antioquia, Medellin, Colombia

**Keywords:** Infectious diseases, Epidemiology

## Abstract

Research on Gestational Malaria (GM) is scarce in America's. In the few available studies in Colombia, the analysis of immunological or parasitological aspects predominates, with few analyzes of epidemiological aspects. The objectives were to determine the frequency of GM and submicroscopic infections (positive with PCR and negative with thick blood smears), to identify obstetric and malaria history associated with GM, and to describe maternal and neonatal outcomes associated with GM, in northwestern Colombia. A retrospective study with records of 825 pregnant women was conducted. qPCR and thick blood smear were performed. Frequencies were determined with 95% confidence intervals. Comparisons were made with the Chi-square test, Mann–Whitney U test, and prevalence ratios adjusted in a log-binomial model. The frequency of GM was 35.8% (95% CI 32.4–39.1) of submicroscopic infection was 16.2% (95% CI 13.7–18.8). According to the multivariable model, the subgroups with the highest frequency of GM were pregnant women without healthcare coverage (32.3%), in the third trimester of pregnancy (30.5%), nulliparous (35.6%), and with a previous diagnosis of malaria in the current pregnancy (64.0%). GM was associated with more frequency of gestational anemia, infection in neonates, and lower birth weight. The results indicate in a precise and direct way that malaria control in this northwestern region of Colombia is far from adequate, which is even more serious considering the affectations for the mother and the neonate.

## Introduction

During the year 2019 around the world, 228 million cases of malaria and 405,000 deaths occured, and 11 million pregnancies were exposed to infection in countries with moderate and high endemicity^1^. Specifically, in America's, there has been an increase in cases since 2015. For example, Colombia registered an increase of 28.2% in 2019, with 66,581 infected^2^. In Colombia, malaria has an unstable pattern characterized by variable interannual and inter-monthly prevalence; it is very common in adults, generally symptomatic, with a wide clinical spectrum (from asymptomatic to fatal); it causes anemia with high frequency; and produces mortality in all age groups^3^.

In Colombia, malaria cause significant outbreaks along the Pacific Coast, the Magdalena and Amazon basins, and is associated with clusters in municipalities of the Pacific Coast, the border with Panama, the north of Antioquia, municipalities of Córdoba, Vichada and Antioquia^6^. The persistence of malaria in Colombia cannot be explained by *Plasmodial* resistance to drugs or resistance of *Anopheles* to insecticides^4–6^. The official reports do not specify the morbidity or mortality cases from gestational malaria (GM); however, with data from the National Institute of Health (NIH) of Colombia, it is possible to say that: (a) in the rural population exposed to malaria in Colombia (living up to 1800 masl in municipalities without adequate antimalarial control) there are 58,752 births, which, we assume, would correspond to the number of pregnant women exposed; (b) based on the data on severe malaria from the NIH 2007–2020, there is 1 case of severe GM for every 2833 total reported cases of malaria; (c) between 2007 and 2020, there were 380 cases of severe/complicated malaria in pregnant women in Colombia, 27.1 cases/year^4,5^.

Beside, GM constitutes a disease with different control and prognosis from malaria in the non-pregnant population. GM produces serious consequences on maternal (anemia, severe malaria, and death), fetal (abortion, anemia, death), neonatal (stillbirth, low birth weight, congenital and neonatal malaria), and infant health (anemia, malnutrition, increased risk of other infections and death)^7–9^. The few studies carried out in Colombia have reported an increased risk of anemia (55.6%); severe malaria with liver dysfunction, acidosis or thrombocytopenia (13.5%); alteration of the development of the immune response to the anti-tetanus toxoid vaccine; and lower birth weight^10–13^.

Concerning the magnitude of GM, most of the evidence is concentrated in Sub-Saharan Africa. In this continent, a meta-analysis with 171 studies between 1990 and 2011 with 340,904 pregnant women found a prevalence of 32% (95% CI 25.9–38.0) in the East and South, and 38.2% (95% CI 32.3–44.1%) in the central and western part of the continent^14^. In America's, studies on the prevalence of GM are meager^15–18^, and particularly in Colombia, there is no clear and precise measurement of the event. In three municipalities of Urabá Antioqueño (Necoclí, Turbo, Carepa), a prevalence of 10.39% was found with thick blood smears (TBS)^12^. In 129 parturient from Turbo-Antioquia and Puerto Libertador-Córdoba, the prevalence of GM was 3.1% with TBS and 14.0% with PCR (*Polymerase Chain Reaction*)^19^. Other studies have reported frequencies of 13% or 14% with TBS^20,21^ versus 32% with PCR, demonstrating a high frequency of submicroscopic GM (positive with PCR, negative with TBS)^21,22^.

The evidence presented reveals several problems that justify the need for more studies on this topic: (a) heterogeneity in the frequency of GM and difficulties in generalizing the available evidence, given that most studies are based on non-probability samples, (b) the low number of studies in Colombia, (c) malaria control programs focused on TBS which generates underreporting and does not detect submicroscopic infections, (d) there are few studies on associated factors to guide other research, and prioritize higher-risk groups, and (e) the primary outcomes of GM on maternal and neonatal health are unknown.

The objectives of this investigation were to determine the general frequency of GM, the specific frequency of its submicroscopic form and of each *Plamosdial* species; to identify the obstetric and malaria history associated with GM; and to describe the principal maternal and neonatal outcomes of GM in the most endemic region in northwestern Colombia, 2009–2020. The human population of the region, during these years, has not registered significant changes in demographic, socioeconomic and cultural characteristics. Malaria data indicate a decreasing trend between March 2010 and March 2014, when the “Colombia Malaria Project” was executed in 43 municipalities with a high endemicity, and it was possible to reduce the cases about 60%, compared to the previous years. Once this Project was finished, the State did not continue it, and malaria increased again, with growing trend until 2020.

### Study location

In 2019, Colombia had 241 municipalities without effective malaria control, especially in rural areas. The rural population residing in these areas with the active transmission is 3,334,116 inhabitants (6.6% of 50.4 million Colombians), located in the five natural regions of Colombia and 21 departments. Based on the adjusted data for births in Colombia in 2019 (702,368 births, according to the National Administrative Department of Statistics, DANE [for its acronym in Spanish]), the population of pregnant women exposed to malaria in 2019 in these 241 municipalities was 58,186 (0.5% of the total rural population). In general, the living conditions of the population residing in these areas with active malaria transmission are very poor and demonstrate great economic poverty^4,5^.

The study region has an extension of 34,848 km, and it is made up of the areas of Urabá Antioqueño, the upper basins of the Sinú and San Jorge rivers, and the Bajo Cauca Antioqueño. The region has had a high malaria incidence (annual parasite index > 25/1000 exposed) since 1950 and generates at least 60% of Colombia's total annual malaria cases. This region comprises 25 municipalities, distributed in the departments of Antioquia (21 municipalities) and Córdoba (4 municipalities). According to official DANE data, the total population of this region was 1,114,000; all of them are a population exposed to malaria, of which approximately 5414 are exposed pregnant women^4,5^.

## Methods

### Study type and subjects

A retrospective study was carried out based on the records of pregnant women who participated in five investigations between 2009 and 2020. The objectives of these investigations were to describe the following aspects of gestational, placental and/or congenital malaria: the parasitic genetic variability, the relationship of placental damage with the expression of cytokines; the immunomodulatory effect of placental *Plasmodial* infection in pregnant women and neonates; the epidemiological and histopathological aspects of placental and gestational malaria; the epidemiology and consequences of submicroscopic *Plasmodial* infection; and the comparison of the outcomes of microscopy, polymerase chain reaction, and histopathology in the diagnosis of the infection. Given that in Colombia this type of research does not receive much money to finance it, at the beginning of these investigations, epidemiological information was collected, in order to accumulate a good amount of data that allowed the development of the current study, for which the same diagnostic tests, observational designs, and epidemiological surveys were applied.

The pregnant women met the following inclusion criteria: stable residents (at least one year before the beginning of each study) of the study region, with TBS and qPCR results for malaria diagnosis, apparently healthy as recorded in their clinical history (without a diagnosis of diseases or infections, or complicated malaria during pregnancy), attend or not the prenatal consultation but have the delivery in any of the public hospitals of the municipalities of the region; with voluntary participation in the study and signing of the informed consent. The research does not carry out any specific activity to promote attendance at prenatal consultation or delivery at the local hospital (the population represents the usual dynamics of delivery assistance in the region, and that it is not a population with possible selection biases). Exclusion criteria were being under antimalarial treatment in the previous 2 weeks, presence of any disease or infection according to the criteria of the health personnel caring for pregnant or laboring women.

In total, the data of 825 pregnant women were analyzed; it corresponds to the following parameters: expected proportion of GM 25%, confidence 95%, sampling error 3%, and estimated population of pregnant women exposed to malaria based on the number of births 15,000, correction of 8%.

### Information gathering

A peripheral blood sample was taken from each pregnant woman by venipuncture. Slides were made for the microscopic diagnosis of malaria by TBS, and Whatman No.3 filter paper circles were blotted for DNA extraction with the Saponin-Chelex method and subsequent molecular diagnosis of maternal *Plasmodial* infection using qPCR^23,24^. In addition, a questionnaire was filled out to extract the epidemiological information from the clinical history, and it contained obstetric information and a history of malaria. The questionnaire was designed by malaria, GM, and epidemiology experts, guaranteeing its appearance (applicability and acceptability) and content validity.

Selection bias was controlled by including all subjects who met the eligibility criteria. Information biases were controlled by training the field team, standardizing the fieldwork, internal quality control in the laboratory, implementation of the test manufacturer's instructions, double data entry, and appearance and content validity of the questionnaire. Confounding variables were analyzed using generalized linear regression.

### Analysis plan

#### General and specific frequency of GM

The general frequency of GM (positive by TBS or PCR) and the specific frequency by type of diagnostic test, as well as the frequency of submicroscopic GM, were calculated, all with their 95% confidence interval. Due to the fact that no statistical differences were found in the frequency by year and municipality of study, the specific frequencies for these two variables were not presented.

#### Obstetric and malarial factors associated with GM

The obstetric variables and malaria history were described with absolute (n) and relative (%) frequencies for categorical variables, and central tendency and dispersion measures for the continuous variables. The possible associations of these variables with GM were explored with Pearson's Chi-square test for nominal variables, Chi-square test for trend for ordinal variables, and Mann–Whitney U test for continuous variables (the normality was determined with Kolmogorov–Smirnov test with Lilliefors correction). The strength of the association was established with prevalence ratios (PR) with the Katz method and its 95% confidence intervals. For polytomous variables, dummies were constructed, taking as a reference group the one with the lowest frequency of GM. A multivariable generalized linear model was performed with the variables that showed a bivariate association with the frequency of GM to identify possible confounding factors. Furthermore, the variables with the most significant explanatory potential for GM were identified through a multivariable generalized linear model with the logarithm transformation and binomial family (log-binomial)^25,26^.

#### Primary maternal and neonatal outcomes related to GM

Among women with and without GM, the frequency of gestational anemia (hemoglobin < 11.0 g/dL), abortions, fetal death, neonatal death, low birth weight (less than 2500 g), and neonatal malaria (umbilical cord blood or blood from the newborn positive for malaria by TBS or qPCR) was compared using Pearson's Chi-square test or Fisher's Exact test (when the expected frequency was less than 5), and the number of abortions, fetal and neonatal deaths, hemoglobin and weight at birth were compared through the Mann–Whitney U test.

All analyzes were performed in SPSS 27.0 with a significance of 0.05.

### Ethical aspects

The guidelines of the Declaration of Helsinki and Resolution 8430 of Colombia for research with pregnant women were applied. All pregnant women signed the informed consent (or assent for those under 18 years of age). The project was endorsed by the Bioethics Committee of the “Sede de Investigación Universitaria SIU”, Minutes # 21-101-961, and was classified as a minimal risk study.

## Results

### Frequency of GM

The frequency of GM was 35.8% (95% CI 32.4–39.1); with TBS was 19.5% (95% CI 16.7–22.3) and the submicroscopic infection was 16.2% (95% CI 13.7–18.8).

### Obstetric and malarial factors associated with GM

The mean time of residence in the malarious area was 8.8 ± 8.2 years (median 7, interquartile range 1–15, range 1–33 years). The mean age of the pregnant women was 23.3 ± 6.4 years (median 22, interquartile range 18–28, range 11–43 years). The average of age gestational in weeks was 30.2 ± 9.8 (median 34, interquartile range 23–39, range 4–43 weeks). The highest proportion of pregnant women were adolescents or young women under 25 years of age (63.8%), primigravidae (31.6%), multiparous (31.2%), 24.2% with previous episodes of malaria during the current pregnancy, 34.0% with malaria in the last year, and 43.7% mentioned the use of mosquito net. Affiliation to the General System of Social Security showed that 93.4% were affiliated to the subsidized regime (Table 1).

**Table 1:** Description of obstetric characteristics and malaria history in the study group.

Variables	Levels	n	%^a^
Healthcare coverage	None	31	5.8
	Subsidized	496	93.4
	Contributory	4	0.8
	Total	531	100
Age group (years)	11–19	269	32.6
	20–24	257	31.1
	25–29	149	18.1
	30–43	150	18.2
	Total	825	100
Trimester of pregnancy	One (week 4–12)	44	6.1
	Two (week 13–26)	211	29.3
	Three (week 27–43)	466	64.6
	Total	721	100
Number of pregnancies	One	261	34.3^b^
	Two	185	24.3
	Three	130	17.1
	Four	84	11.0
	Five	47	6.2
	Six to thirteen	54	7.1
	Total	761	100
Number of births	Nuliparous (0)	222	35.1 ^b^
	Primiparous (1)	153	24.2
	Multiparous (2–10)	257	40.7
	Total	632	100
Malaria during current pregnancy	Yes	200	26.3
	No	561	73.7
	Total	761	100
Malaria in the last year	Yes	281	50.4
	No	277	49.6
	Total	558	100
Treatment of the last malaria	Yes	125	44.5
	No	91	32.4
	She does not remember	65	23.1
	Total	281	100
Use of mosquito net	Yes	361	63.2
	No	210	36.8
	Total	571	100

^a^The percentage was calculated based on the total number of observations registered in each variable (data lost or not registered in the medical record were excluded).

^b^These frequencies should be the same, but the medical history does not make an exhaustive record of the number of abortions.

The frequency of GM only showed association with the number of deliveries, previous episodes of malaria during the current pregnancy, and malaria in the last year, being 83% higher in nulliparous women compared to multiparous, 3.29 times more in pregnant women with a previous diagnosis during the current pregnancy and 4.24 times more in those who suffered malaria in the last year compared to those who did not have this diagnosis (Table 2). In the multivariable analysis, the diagnosis of malaria in the last year was not statistically significant, indicating that its bivariate association was the product of a confounding effect.

**Table 2:** Specific frequency of gestational malaria, prevalence ratio, and odds ratio, according to obstetric characteristics and malaria history.

Potential associated factors	Frequency % (n)^a^	PR (CI 95%)	p
**Healthcare coverage**			
None	32.3 (10)	1.33 (0.78–2.27)	0.312
Subsidized^b^	24.2 (120)		
**Age group (years)**			
11–24	37.6 (198)	1.16 (0.95–1.41)	0.139
25–43^b^	32.4 (97)		
**Gestation trimesters**			
One^b^	18.2 (8)	1.0	
Two	28.9 (61)	1.59 (0.82–3.08)	0.145
Three	30.5 (142)	1.67 (0.88–3.18)	0.087
**Pregnancies**			
One	32.6 (85)	1.07 (0.84–1.36)	0.590
Two	31.4 (58)	1.03 (0.78–1.35)	0.838
Three or more^b^	30.5 (96)	1.0	
**Deliveries**			
Nulliparous	35.6 (79)	1.83 (1.34–2.48)**	< 0.001**
Primiparous	26.8 (41)	1.37 (0.96–1.98)	0.087
Multiparous^b^	19.5 (50)	1.0	
**Malaria in the current pregnancy**			
Yes	64.0 (128)	3.29 (2.70–4.01)**	< 0.001**
No^b^	19.4 (109)		
**Malaria in the last year**			
Yes	50.5 (142)	4.24 (3.02–5.96)**	< 0.001**
No^b^	11.9 (33)		
**Treatment of the last malaria attack**			
No	46.2 (42)	1.07 (0.79–1.44)	0.666
Yes^b^	43.2 (54)		
**Use of mosquito net**			
Yes	26.6 (96)	1.21 (0.89–1.65)	0.211
No^b^	21.9 (46)		

*p < 0.05. **p < 0.01. *95% CI* 95% Confidence interval.

^a^Proportion of positives in each subgroup, the total number of subjects in the subgroup indicated in each row is taken as the denominator

^b^Reference group or denominator of the PR (Prevalence ratio).

The following variables had the major explanatory potential for the frequency of GM: healthcare coverage (higher in pregnant women without coverage), trimester of pregnancy (higher frequency in the third trimester), number of deliveries (higher in nulliparous women) and previous diagnosis of malaria in the current pregnancy (Table 3).

**Table 3:** Multivariable generalized linear model to identify potential explanatory factors for the frequency of gestational malaria.

	PR (CI 95%)	Wald Chi-square	p
Healthcare regime (none/subsidized)	2.34 (1.00–5.94)*	5.4*	0.041*
**Trimester of pregnancy**			
Second/first	1.65 (0.60–4.51)	1.6	0.301
Third/first	3.23 (1.75–5.94)**	14.9**	< 0.001**
**Deliveries**			
Nulliparous/multiparous	2.53 (1.00–8.77)*	6.4*	0.038*
Primiparous/multiparous	1.95 (0.77–4.89)	2.4	0.252
Malaria in current pregnancy (yes/no)	8.90 (6.3–25.0)**	48.4**	< 0.001**

*p < 0.05. **p < 0.01. *PR* Prevalence ratio, *95% CI* 95% Confidence interval.

### Primary maternal and neonatal outcomes related to GM

GM was related with anemia, being 87% higher in women with GM compared to pregnant women without malaria; and higher frequency of malaria in neonates, being 3.91 times higher than what was found in pregnant women without malaria. Although low birth weight did not present a statistical association with GM, a statistically lower median birth weight was found in pregnant women with malaria than in pregnant women without the infection (Table 4).

**Table 4:** Comparison of maternal, fetal, and neonatal outcomes in pregnant women with and without malaria.

Outcomes	Total		With GM (n)	Without GM (n)	PR (CI 95%)	p
	N	(n)				
Gestational anemia	527	32.1 (169)	46.4 (83)	24.7 (86)	1.87 (1.47–2.39)**	< 0.001**
Abortion	568	15.5 (88)	12.8 (18)	16.4 (70)	0.78 (0.48–1.26)	0.302
Fetal death	289	1.0 (3)	2.9 (2)	0.5 (1)	6.50 (0.60–70.6)	0.102
Stillbirth	565	2.5 (14)	3.6 (5)	2.1 (9)	1.69 (0.57–4.94)	0.322
Low birth weight	620	6.5 (40)	7.1 (14)	6.2 (26)	1.15 (0.61–2.15)	0.672
Neonatal malaria	443	11.5 (51)	27.3 (27)	7.0 (24)	3.91 (2.36–6.46)**	< 0.001**

	Median (interquartile range)			p U de M-W		
Gestational hemoglobin	11.4 (10.6–12.3)	11.0 (10.0–12.0)	11.6 (11.0–12.4)	< 0.001**		
Birth weight (kg)	3.1 (2.8–3.4)	3.0 (2.8–3.4)	3.2 (2.9–3.4)	0.009**		

*PR* Prevalence ratio, *95% CI* 95% confidence interval, *M–W* Mann–Whitney.

*p < 0.05. **p < 0.01.

## Discussion

The frequency of GM was high (35.8%) with many cases of submicroscopic infection (16.2%), which aggravates the situation, because the diagnostic test of malaria used routinely in the area is TBS. Gestational anemia, neonatal malaria and birth weight are significantly associated with presence of GM. These data show several relevant aspects to study GM: (a) its high frequency, which implies a high risk for maternal, fetal, neonatal, and infant health; (b) very high endemic levels despite reports from national authorities indicating that malaria is a controlled infection at the beginning of the pre-elimination stage^27^; (c) higher risk of infection in some population subgroups that should be prioritized (pregnant women without health coverage, third trimester of pregnancy, nulliparous and with previous diagnosis of malaria); and (d) high heterogeneity in the frequency of GM according to the presence of some associated factors, which shows the importance of conducting local studies that allow a deeper understanding of clinical, epidemiological, cultural, and socioeconomic conditions that increase the risk of developing the disease^28,29^.

The frequency of GM was 35.8% (95% CI 32.4–39.1), which is statistically the same as the one reported in a meta-analysis of 171 studies with 340,904 pregnant women treated in prenatal care programs in sub-Saharan Africa, where the frequency was 32% (95% CI 25.9–38.0) in the East and South part of the continent, and 38.2% (95% CI 32.3–44.1%) in the central and west part of the continent^14^. Other studies from America's have documented smaller proportions than the current study. In 449 pregnant women from the state of Bolívar-Venezuela, it was 27.4%^17^ and in a study in Brazil with data from the passive surveillance system, it was 7.5%; however, in this latter case, the low proportion was attributed to the absence of an active search for cases and the lack of registration of asymptomatic and submicroscopic cases, which constitutes a clear detection bias^18^. In the specific case of Colombia, previous studies have reported frequencies of GM ranging from 10.4% with TBS to 32% with qPCR^12,20,21^. In a meta-analysis of the prevalence of malaria associated with pregnancy in Colombia 2000–2020 the prevalence of GM was 5.8% (95% CI 3.8–8.7) by TBS, 16.7% (95% CI 9.0–28.8) with PCR, and 8.5% (95% CI 3.4–19.7) of submicroscopic infections; with a high heterogeneity in the magnitude of the infection, attributable to the level of endemicity of the different regions studied, and variations in the study designs^30^. The high frequency reported in different populations, shown the high gynecological-obstetric risk attributable to GM, and it indirectly demonstrates problems in the control of the infections in prenatal care programs. This situation is aggravated when considering that among the pregnant women of this research, some subgroups exceeded these frequencies and, therefore merit priority actions in healthcare, control, and prevention.

Also, it is essential to emphasize the high frequency of submicroscopic infection (16.2%; 95% CI 3.7–18.8), which has been documented in previous studies^21,22^, demonstrating greater risks to the health of the mother–child pair since this type of infection is not treated, which means that it can progress to cases of severe fetal, congenital or neonatal malaria, and other consequences mentioned in the introduction^7–12^. Previous studies in the same endemic region confirm the pathological ability of microscopic or submicroscopic infections to produce malaria in the mother, the placenta, and the neonate^31–33^. Some researchers refer beneficial aspects of persistent and repeated submicroscopic malaria; this has been described for cases of asymptomatic malaria (without specifying whether they are submicroscopic cases) associated with decreased incidence of malaria, but without protection against disease when new infection occurs^34^. Other studies show otherwise, since persistent asymptomatic *P. vivax* and *P. falciparum* infections, with low-density parasitaemias, produces higher density infections at a later time, with the subsequent clinical outcomes^35^. Other authors have indicated that the evidence available in this topic does not show beneficial to the individual, even, this should be named as chronic malaria infections, which is a construct with major scientific, operational, and ethical challenges^36^. It is crucial to detect and treat submicroscopic malaria to control endemicity, disrupt transmission, and to target all malaria infections, irrespective of their density or presentation^35–37^.

The standard test applied in Colombia's endemic areas is TBS, whose diagnostic ability is low compared to qPCR, which leads to a high proportion of false negatives. Added to this is the high number of pregnant women who live in remote areas and are not detected. For this reason, it is crucial to improve diagnostic efforts, especially considering that in Colombia, the high cost-effectiveness of some strategies for GM diagnosis has been demonstrated^38^.

The multivariable analysis identified the following explanatory factors of GM: healthcare coverage, trimester of the pregnancy, number of deliveries, and previous diagnosis of malaria in the current pregnancy. These findings coincide with other studies that have identified as the main risk factors for GM the weeks of gestation and previous diagnosis of malaria^12,39^, without association with gravidity^18^; while it differs from other that have reported the association with having a higher number of pregnancies; although in this last study the association was detected in a bivariate analysis, without adjusting for possible effect modifying variables in a multivariate analysis^12^. At this point, it is essential to clarify that the explanatory factors found in this research are difficult to contrast with the available literature for several reasons:

- Most studies focus on parasitological or diagnostic variables, without obstetric factors^19–21^.
- The most of observational studies in GM has been done in Africa with other independent variables (ethnic group, marital status, household density, prepregnancy body mass index, prepregnancy mid-upper-arm circumference, and others) and where the obstetric factors are not comparable with the current study population^39^.
- The majority of studies investigating factors associated with GM take the frequency of malaria as independent variables for different obstetric outcomes, which means that the GM effects and not its triggers are studied^40^.
- The studies on risk factors generally compare what happens in pregnant women versus non-pregnant women, which is different from the design and population of the current study^41^.

Despite the limited comparison level of the explanatory factors of GM in this research with the previous literature, this type of multivariable analysis shows the importance of identifying central subgroups for targeting disease prevention, care and healing actions.

The frequency of GM was related to a higher frequency of anemia, infection in newborns, and lower birth weight, which is consistent with the available evidence in this field^7–9^ and with the few previous reports from Colombia^10–12^. However, it is essential to clarify that no association was found with other events related to GM, such as abortion, fetal death, and stillbirth. Abortions presented a high frequency in pregnant women with GM and those without the infection, which indirectly shows the need to investigate other causes of this problem. The presence of fetal death was higher in pregnant women with GM; however, this difference was not statistically significant. Considering that the statistical power in this comparison was 80%, it can be stated that in the South of Córdoba-Colombia, GM is not related to this type of mortality, exposing to the need to investigate other causes. In pregnant women with GM, a higher frequency of stillbirth was also found, without registering a statistically significant difference, but in this case, the statistical power of the comparison was 54%, so it is not possible to rule out malaria as a cause of this problem in the study group.

Added to the above, there are other problems for the control of GM, analyzed in other investigations, such as the nonspecific clinical presentation, insufficient facilities for an efficient diagnosis, lack of trained personnel, absence of clear regulations for mass implementation of diagnostic tests, and low allocation of financial resources for diagnosis and control programs^42^.

Among the limitations of this study, some are attributable to the type of study, such as the exploratory nature of the associations (which do not show causality). Others are related to the information sources used by the Colombian health system that are not exhaustive for all factors associated with GM, and structural problems in the study area where poverty, armed conflict, and other social phenomena prevented an approach with greater external validity. A favorable aspect to highlight of this work is having gathered all the investigations carried out by this research group in 12 years, which allowed us to obtain 825 records, which means a large sample, all of them coming from prospective investigations.

## Conclusion

A high frequency of GM, especially submicroscopic, was found. GM increased the frequency of gestational anemia and infection in neonates and reduced birth weight. The results clearly, precisely, and directly indicate that malaria control in this northwestern region of Colombia is far from adequate, which is even more serious if it is considered that those affected by GM are both the mother and the neonate.

## Data Availability

All data generated or analysed during this study are included in this published article.
